# An Iterative and Collaborative End-to-End Methodology Applied to Digital Mental Health

**DOI:** 10.3389/fpsyt.2021.574440

**Published:** 2021-09-23

**Authors:** Laura Joy Boulos, Alexandre Mendes, Alexandra Delmas, Ikram Chraibi Kaadoud

**Affiliations:** ^1^Saint-Joseph University, Beirut, Lebanon; ^2^Groupe onepoint, Paris, France

**Keywords:** digital mental health, an end-to-end methodology, human factors, cognitive biases, machine learning, knowledge discovery data base (KDD), interdisciplinar intersectoral collaborations, ethics

## Abstract

Artificial intelligence (AI) algorithms together with advances in data storage have recently made it possible to better characterize, predict, prevent, and treat a range of psychiatric illnesses. Amid the rapidly growing number of biological devices and the exponential accumulation of data in the mental health sector, the upcoming years are facing a need to homogenize research and development processes in academia as well as in the private sector and to centralize data into federalizing platforms. This has become even more important in light of the current global pandemic. Here, we propose an end-to-end methodology that optimizes and homogenizes digital research processes. Each step of the process is elaborated from project conception to knowledge extraction, with a focus on data analysis. The methodology is based on iterative processes, thus allowing an adaptation to the rate at which digital technologies evolve. The methodology also advocates for interdisciplinary (from mathematics to psychology) and intersectoral (from academia to the industry) collaborations to merge the gap between fundamental and applied research. We also pinpoint the ethical challenges and technical and human biases (from data recorded to the end user) associated with digital mental health. In conclusion, our work provides guidelines for upcoming digital mental health studies, which will accompany the translation of fundamental mental health research to digital technologies.

## Introduction

### Digital Health Definition

Digital health can be defined as the concept of healthcare meeting the Internet (1). It ranges from telehealth and telecare systems (2) to patient portals and personal health records (3, 4), mobile applications (5), and other online platforms and devices. However, and as opposed to digitized versions of traditional health approaches, digital health interventions (DHIs) (6) utilize artificial intelligence (AI) algorithms and other machine learning (ML) systems to monitor and predict symptoms of patients in an adaptive feedback loop (7). Improvements in ML over recent years have demonstrated potential within a variety of diseases and medical fields including neurological and mental health disorders (8) both at an individual-patient level and applied to larger populations for scalable understanding, management, and intervention of mental health conditions in different cohorts and various settings (7). In addition, and because to our knowledge, effective coverage does not exceed 50% in any country and is much lower in low- and middle-income countries, DHIs also address social problems in the healthcare system such as poor access, uncoordinated care, and increasingly heavy costs (9). Digital mental health interventions could thus give much needed attention to underresearched and undertreated populations (10).

### Digital Mental Health Technology Advances

The keywords “digital mental health” in PubMed's search engine (accessed April 2020) show that 2019 has the largest number of published articles compared to any prior year. The trend is also rising for the keywords “mental health mobile apps,” providing evidence that interest in both (i) publication of articles about digital health and (ii) technical advances is rising. Advances in digital health technologies in mental health are occurring at a rapid pace in research laboratories both in academic institutions and in the industry (11). The rapidly growing number of biological devices and the exponential accumulation of data in the mental health sector aim at facilitating the four purposes of healthcare: diagnosis, monitoring, treatment, and prevention (1).

#### For Diagnosis

Important digital health interventions for characterization or diagnosis include algorithms for illness detection and classification (11). One digital tool that is further revolutionizing mental healthcare is conversational AI (12). Although the clinician–AI collaborations have yet to be specified and the cognitive biases considered (see *Designing digital health systems with human factors approach*), a blended approach (in an AI-delivered human-supervised model) (12, 13) is alluring.

#### For Monitoring

The use of data generated by personal electronic devices to monitor mental health parameters may result in useful biobehavioral markers that could in turn optimize diagnosis, treatment, and prevention and a global clinical improvement (14). This has led to the conception of all sorts of wearable devices and connected objects such as smart watches to collect data in healthy and pathological populations in a scalable unobtrusive way (15, 16), smart textiles to collect and monitor physiological outcome measure such as in athletes (17), or smart homes to monitor biophysiological measures of older people (18). This has also led to the development of various mobile applications (linked or not to a wearable device) that monitor given behaviors or cognitions in specific populations. This is the case of *eMoods*, a mood tracking app conceived for patients with bipolar disorders to follow their fluctuations. This is also the case of *PROMIS*, a mobile application to self-report different cognitive, emotional, and mood measures (19).

#### For Treatment

Beyond diagnosis and monitoring allowed mainly by data interpretation, some digital mental health interventions include assisting and treatment options (1). This is particularly timely as the Food & Drug Administration (FDA) has just approved its first prescription video game in mental health for kids with ADHD: *EndeavorRx* (20).

While digitized versions of classical clinical approaches propose digital conversational agents such as chatbots that provide coaching and cognitive behavioral therapies in a conceptually similar value than a human healthcare provider (7, 21), AI-based algorithms and data-driven digital health initiatives further aim at implementing more adaptive algorithms and flexible, personalized treatments *via* AI and ML (8, 21). Such is the case of *Open Book*, an assistive technology tool for adaptive, personalized text simplification for people with autism spectrum disorder (22). It is also the case of *Entourage*, a novel digital intervention that improves social connection for people with social anxiety symptoms (23), or *Doppel*, a device that helps people manage their daily stress by modulating physiological and emotional states through a heartbeat-like rhythm tactile sensation (24). Other digital mental health interventions for treatment purposes include virtual reality-based exposure [in the treatment of anxiety disorders for instance (25)] as well as the use of robotic technology [to improve social interactions in people with dementia for instance (26)].

#### For Prevention

By opening new modes of real-time assessment [through longitudinal data collection or through the presence of sensors in smartphones for instance, to track sleep, movement, speech… (27, 28)], digital mental health interventions enable catching new episodes of a given disorder at a very early stage. It is especially the case for suicide preventions (29).

### The Need to Homogenize R&D Processes

In contrast, there is only scarce clinically significant outcomes of digitalized solutions. Although both advances in fundamental research and technical innovations are occurring rapidly, translation from one to the other has been slower (11). This can be explained by the lack of better-designed clinical trials and the loss of interest at the patient level in digital health products over time, both of which lead to poor long-term data and scarce information on whether new behavior facilitated by a digital health tool is long-lasting (30).

Another major problem at the time is the disparity of research and development processes across fields and sectors. One way of accelerating the potential benefits of digital mental health interventions and optimizing the transformation of fundamental discoveries into innovative digital technologies applied to routine clinical practice would be to propose a methodology that could be used across disciplines and sectors in the field of mental health. This would include homogenizing research and development processes in academia as well as in the private sector; improving technical methods that standardize, aggregate, and exchange data; centralizing data into federalizing platforms focusing on scalability; and establishing data repositories, common data standards, and collaborations (14, 31).

### The Global Pandemic Context

In March 2020, the WHO declared the novel coronavirus disease of 2019 also known as COVID-19 as a global pandemic. Today, a year later, the WHO counts 185,038,214 confirmed cases of COVID-19 globally, including 3,250,648 deaths. Amid this rapidly evolving sanitary crisis, digital innovation is being used to respond to the urgent needs of the pandemic. Actions in the field have been involving multiple stakeholders, from frontline healthcare to public health and governmental entities. They have also raised new challenges regarding the link between academia and the industry, the different velocities at which the two sectors evolve, the ethical questions of data collection, and the various geographical and socioeconomic inequalities due to limitations in capacity or resources (32).

Apart from the direct risks of COVID-19 on health and the healthcare system, the uncertainty of the context and the high death rate due to the virus also exacerbate the risk of mental health problems and worsen existing psychiatric symptoms, further impairing the daily functioning and cognition of patients (33).

While these illnesses do not all represent an immediate threat to life, they will have long-lasting serious effects on individuals and large populations. Emerging mental health issues should thus be addressed promptly. In addition, the multiple logistic changes imposed on us by the pandemic pose a unique challenge in mental health service delivery. For example, the restriction in freedom of movement and face-to-face therapies increases psychological distress (32). The limited knowledge on the virus and the overwhelming news that surround it also increase anxiety and fear in the public (33, 34). In addition, long quarantine durations are generating frustration, boredom, stigma, and stress, as well as financial loss that also affects mental health. This is without mentioning highly vulnerable populations such as healthcare providers (32), university students (35), children (36), and naturally anxious individuals (37) who are more prone to developing mental illnesses such as posttraumatic stress disorder or anxiety and mood disorders during this pandemic crisis. This is also without mentioning the already.

In this context and with the advent of AI, a digital methodology that optimizes and homogenizes research processes in an intersectoral and transdisciplinary approach makes more sense than ever, specifically in the field of mental health. Implementing such approaches could help detect and monitor mental health symptoms and their correlation to COVID-19 parameters (whether individuals are affected by the virus or know people affected by it, how political decisions impact mood and anxiety of general populations, etc.). Early detection and close monitoring would in turn allow adequate in-time treatment in the short term and prediction as well as prevention in the longer term.

### Introduction to Our Work

Here, we propose an end-to-end methodology that highlights key priorities for optimal translational digital mental health research. Each step of the process is elaborated from brainstorming to product creation, with a focus on data analysis. Based on iterative processes, the methodology aims at being cross-sectorial, at the intersection between academia and the private industry. By formalizing the methodology around a mental health use case, the methodology also aims at being interdisciplinary, encompassing different fields (from computational neuroscience to psychology and well-being) all while stressing on the importance of human factors in the digitalization of health. An important goal of the methodology is thus to allow robust collaborations between experts from different fields and sectors (practicing clinicians, AI researchers in academic institutions, and R&D researchers in private industries) to pinpoint then advance foundational and translational research relevant to digital mental health and to create ultimately digital tools that satisfy various stakeholders (usability, clinical benefit, economic benefit, security, and safety). All in all, our methodology has the short-term ambition to propose guidelines for upcoming digital mental health studies and the ultimate ambition to transform the gap between fundamental and applied research into a federalizing platform.

## Conceptualization and Project Learning

### Project Idea and Concept

#### Evaluating the Feasibility of an Idea

All research begins with a question. Not all questions are testable though, and the scientific method only includes questions that can be empirically tested (observable/detectable/measurable) (38). Similarly, not all questions lead to the development of solutions. As a matter of fact, only few research projects directly reach practical solutions. However, in the digital health sector, research tends to have (or at least ought to have) a very pragmatic, concrete, and measurable outcome (39). The selection of ideas is therefore one of the most complex steps of the research process in the digital health sector since, in addition to verifying whether their idea can be transformed into a project, researchers must also evaluate whether the project can lead to practical often technical solutions, and when. As the world of technologies moves fast (11), by the time that an idea leads to a solution, the solution might lose some or all its value. It is thus crucial to assess whether the idea is feasible and realistic early in the process.

As a result of the COVID-19 pandemic context for instance, there has been an increase in the usage of telehealth medicine and alternative digital mental health options such as mobile applications and web-based platforms (40). Although the need is real and measurable, research projects must be cost-effective, and depending on the investment needed, they ought to be useful not only within a short-term period (i.e., to treat current psychiatric illnesses) but also in a longer time frame, to treat for instance the expected rise in symptoms of trauma among the general population (40).

**Figure 1:**
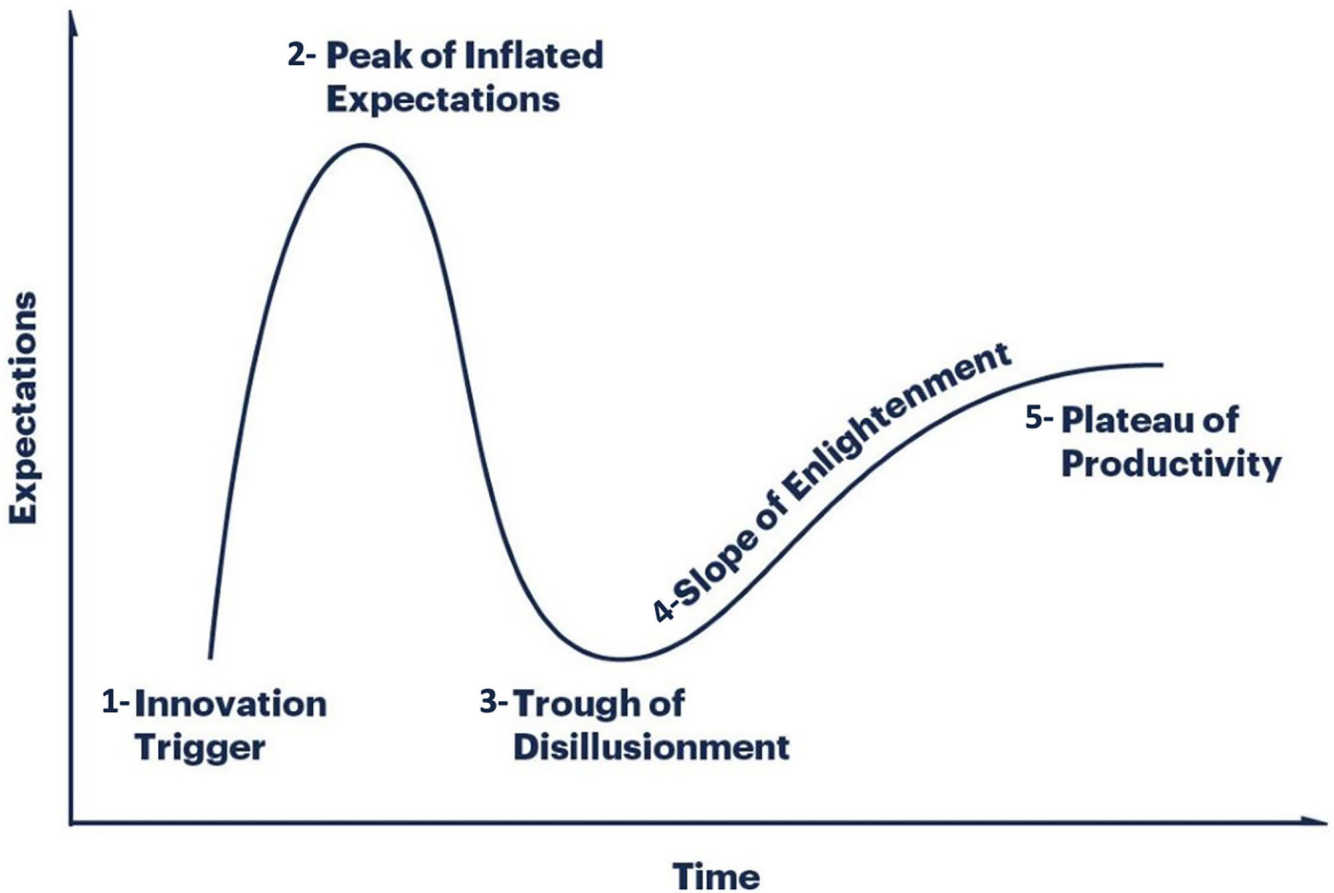
The five stages of the hype cycle of digital health research: (2) the innovation trigger, (3) a peak of inflated expectations, (4) a trough of disillusionment, (5) a slope of enlightenment, and (6) a plateau of productivity. Adapted from Gartner (41).

In addition, according to Gartner (1), digital health research follows a hype cycle divided into five stages illustrated in Figure 1:

1. the innovation trigger,
2. a peak of inflated expectations,
3. a trough of disillusionment,
4. a slope of enlightenment, and
5. a plateau of productivity.

An ideal digital health research project predicts the failures that will occur at the third stage and the plateau that will be reached at the fifth stage in order to prepare for them and increase the chances of overcoming them. For instance, treating traumas through mobile applications might 1 day be the old-fashioned way of approaching such disorders. This is one major challenge as there are no generic methods describing a digital health project from research inception to solution development (1).

Evaluating an initial idea further faces more classical challenges such as finding the good mix between focused enough to be interesting yet broad enough to build on existing knowledge (42). A digital health research project should balance more than any other research project between ambitious but not overambitious as the competitive landscape is both wide and niche. Going back to our COVID-19 example, this would mean developing digital health technologies that are precise enough to treat specifically traumas in a pandemic context, but broad enough to be adaptable when traumas would not be the main mental health issue anymore, in a near enough future.

#### Defining the Goal and the Approach

What is it that we want to put in light? Defining the goals and objectives of a digital health research project is essential as it keeps the project focused (11). The process of goal definition usually begins by writing down the broad and general goals of the study. As the process continues, the goals become more clearly defined and the research issues are narrowed to an extent that depends on the adopted approach. For instance, the general goal of a mental health mobile application could be to improve mental health conditions; this is the case of the 1,009 psychosocial wellness mobile apps that were found in a study looking to differentiate scientifically evidenced apps from the success stories due to a media buzz (43). A more palpable goal could be to promote behavioral change; this is the case of *notOK*, a suicide prevention application that alerts the support system of a patient when negative thoughts are too close to an acting out. This is also the case of *Twenty-Four Hours A Day*, an addiction app that offers 366 meditations (one per day) to help abstinent patients focus on sobriety. The goal ought, however, to be further narrowed as the design of the application might consider eliciting not only more engagement on the mobile app overall, but perhaps effective engagement defined by specific patterns (44). *Twenty-Four Hours A Day* could, for example, be used effectively during a year at the end of which users could lose interest, potentially resulting in a relapse. Narrowing the number of users could allow a deeper engagement of actual users; more is not always better in digital health (45).

Given the multistakeholder nature of healthcare and their varying incentives, the best approach to impactful and useful digital health research may differ depending on the project. The main challenge is to find the right balance that maximizes clinical impact all while utilizing efficient resources and at a rate that corresponds to the needs of the market in a globally very dynamic and rapidly changing digital health landscape. This brings us back to creating a requirement set broad enough to encapsulate concepts important to all products, but not too inclusive that the requirements are not relevant anymore (39).

This also allows us to emphasize on the importance of staying flexible and ready to change strategies depending on the number and the rate at which new technical solutions are deployed with time. *Headspace* for instance had started as an events company organizing mindfulness trainings and workshops; as they stayed open to opportunities, they later developed their mobile application that is currently being used in several clinical trials (46). In the case of this app, adopting a ready-to-change pivot strategy allowed them to seize an opportunity and scale drastically.

One way to stay flexible is to inject some agility in the research processes. Agility uses iterations (also called sprints) to create short loops of work (1–4 weeks) that start with planning and end with retrospection, favoring more frequent deliverables (such as quick posters or abstract publications, proof of concepts or minimum viable products) (47). If the concept of agility springs from the software development field, it has been more broadly applied in different fields and sectors recently, such as in mobile health technology (47). A clear step-by-step example applied to our use case, i.e., digital mental health, is the text-based coaching practical guidance provided by Lattie et al. (48).

All in all, it is crucial to define then narrow the goal progressively while balancing between clinical requirements and market realities by staying agile and considering the potential conceptions and misconceptions of all stakeholders.

#### Clarifying Digital Health Research Conceptions and Misconceptions

Everyone is susceptible to the misconceptions of research, development, and innovation, including researchers and any other individual in academia or in the private industry (see *Identifying cognitive biases in digital health to improve health outcomes*). The what of research is challenging in itself and even more so in the digital health context that often includes translational application at the end of the process as well as the need to confront the views and requirements of academia and the industry. It is therefore critical to identify these misconceptions early in the research project to reduce them and promote alternative conceptions where necessary. Most common misconceptions include the following (49):

- Good research procedures necessarily yield positive results.
- Research becomes true when published.
- Properly conducted research never yields contradictory findings.
- It is acceptable to modify research data to make them look perfect.
- There is only one way of interpreting results.

Discussing conceptions and misconceptions of research can reduce cognitive biases (see *Identifying cognitive biases in digital health to improve health outcomes*) and improve research outcomes all while favoring a holistic approach to research (42):

- How would you describe research to your grandmother?
- What is the difference between academic (moving knowledge further, contributing to the development of the discipline, explaining, arguing, conceptualizing, theorizing, developing insights, being rigorous and methodical, situated within a theoretical or conceptual tradition) and industrial research (fact-finding, collecting and reporting, producing and developing)?
- How to combine different views and different approaches and methods of research into an R&D model that serves research and innovation in the digital health sector?

This “awakening” step is of particular importance in DHI as interdisciplinary and intersectoral collaborations increase by the day (see *Identifying the team and potential partners or collaborators*).

#### Extending the Literature to a Market Research

Reviewing the literature is an inevitable step of a research project (for further details, see Appendix 1). Nonetheless, it cannot factor in major advances in health technology if relying only on peer-reviewed sources (50). Given both the size (valued at 75 bn in 2017 by Technavio's Global Digital Health Market research report) and the evolution rate (projected to reach 223 bn in 2023 as predicted by Global Market Insights) of the digital health market, it seems crucial to complement the literature review with adequate market research also called gray literature.

Given the complexity that is characteristic of the digital health landscape of technologies, market research cannot be straightforward. For it to be as thorough as possible, it should include project reports, market research foresees, policy documents, and industry white papers (39). For instance, in the oversaturated market of mobile apps advocating for wellness and self-care, one approach would be to conduct a systematic review of publicly available apps on the stores using key words related to the topic (43).

In the context of digital mental health research, the market research would allow researchers not only to compare the potential outcome of their research to the state of current technology (51) but also to predict or at least speculate whether their solution will still have the same value by the time it reaches the market. Such market research could also provide researchers with an overview of the general landscape, i.e., of the unexplored new market areas (blue ocean strategy; 47).

#### Identifying the Team and Potential Partners or Collaborators

Common benefits to collaboration including brainstorming, division of labor, and speed of execution are challenged by the difficulty of developing a shared vision and defining roles and responsibilities for the different collaborators (52). These challenges are exacerbated in the context of digital health as the field is essentially both interdisciplinary and intersectoral (53), bringing together academic researchers, private industries and their R&D departments, clinicians, patients, and other healthcare consumer groups (54). Indeed, while collaborations in the field are facilitated by complementary roles, authentic communication between partners, and clearly outlined goals or expectations prior to the collaboration, they can also be jeopardized by misaligned expectations, differences in productivity timelines, and balancing business outcomes vs. the generation of scientific evidence (53). It is thus crucial not only to identify the right fit for a collaboration but also to outline and communicate openly about goals, expectations, and timelines. This was done by *X2AI*, a US-based digital health company that developed in collaboration with experts (including clinical, ethical, technical, and research collaborators) an ethical code for startups, labs, and other entities delivering emotional AI services for mental health support (55). Once the project is developed, it moves to the commitment phase or project planning.

### Project Execution

#### Sampling

The rapid advancement of digital health technologies has produced a research and development approach characterized by rapid iteration, often at the expense of medical design, large cohort testing, and clinical trials (39, 43). According to the WHO's guidance for digital health research (56), digital research measures are too often evaluated in studies with varying samples and lack of or poor validation. Additional challenges with digital health research include a potentially unrepresentative sample (57). Consequently, insufficient sample sizes may make it difficult for these data to be interpreted through ML techniques (58) (see *Data postprocessing: visualization and evaluation*). Underestimation occurs when a learning algorithm is trained on insufficient data and fails to provide estimates for interesting or important cases, instead approximating mean trends to avoid overfitting (59).

It is, however, necessary to pursue the adequate amount of evaluation and verification to avoid dubious quality and ensure usefulness and adequacy of the solution (60). To do so, it is crucial to improve sampling strategies by including underrepresented groups in the recruitment, collecting and analyzing reasons for declining, analyzing the profiles of recurrent participants (61), and creating ultimately novel smart sampling approaches (62).

#### Choosing the Appropriate Material and Method

Research projects in the digital health sector can take the form of cohort studies, randomized trials, surveys, or secondary data analysis such as decision analyses, cost-effectiveness analyses, or meta-analyses. To sum things up, there are three basic methods of research:

1. *Surveys* by e-mail, *via* a web platform or *via* a mobile application. They usually involve a lengthy questionnaire that is either more in-depth (usually by email) or more cost-effective (web- and app-based surveys) (63).
2. *Observation* monitors subjects without directly interacting with them. This can be done either in the environment of the subject with different monitoring devices (ecological environment) or in a lab setting using one-way mirrors, sensors, and cameras to study biophysiological markers or behavior (controlled environment) (64). Faster digital tools now allow monitoring patients *via* their health insurance or *via* different health apps.
3. *Experiments* allow researchers to modify variables and explain changes observed in a dependent variable by a change observed in the independent variable. Experiments were mostly restricted to laboratory contexts as it is very difficult to control all the variables in an environment. This contextual limitation is, however, blurred with digital health research and the use of technologies in less controlled environments. In addition, and even within a laboratory, attention should be given to hardware and software variability between devices as it can affect stimulus presentation and perception of a stimulus as well as human–machine interaction (64).

Although there is no one best method for all digital health research projects, a well-defined problem usually hints at the most appropriate method of research. There also often are cost/quality trade-offs that urge the researcher to consider budget and time as part of the general design process.

## Project Design

### Designing Digital Health Systems With Human Factors Approach

#### What Is User-Centered Design?

**Figure 2:**
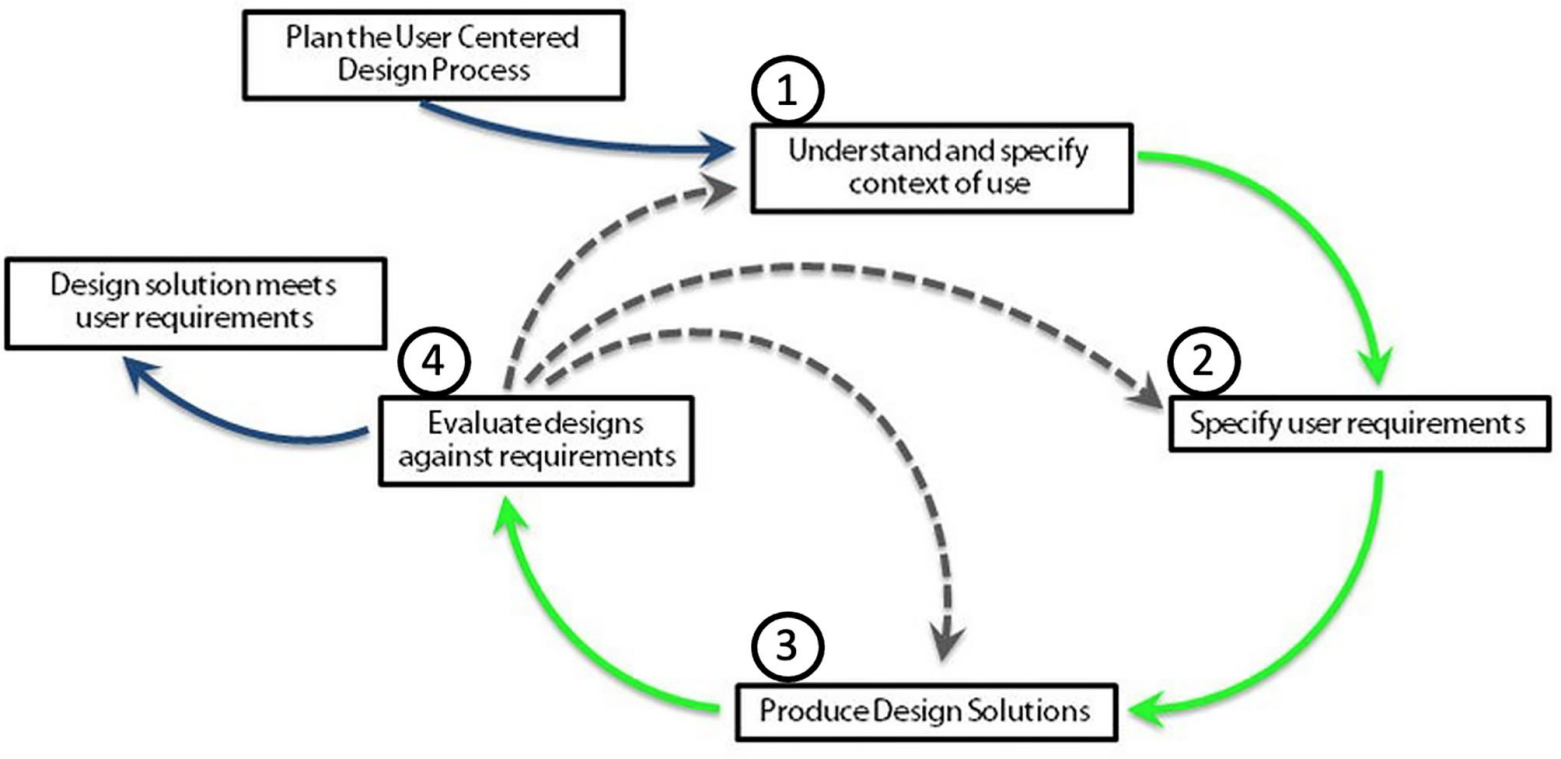
The four phases of the user-centered design: (2) understanding the context of use, (3) identifying and specifying user requirements, (4) designing solutions, and (5) evaluating the outcomes of the design to assess its performance. Adapted from Nielsen (65).

A user-centered design (UCD) is an iterative design process in which designers focus on users and their needs in each phase of the design process. Design teams may include professionals from multiple disciplines (ethnographers, psychologists, engineers), as well as domain experts, stakeholders, and the users themselves. They also involve users throughout the design process *via* a variety of research and design techniques (surveys, interviews, brainstorming), to create highly usable and accessible products. Each iteration of the UCD approach involves four distinct phases illustrated in Figure 2 (65) [see norm ISO (9241-210, 2010)]:

1. understanding the context of use,
2. identifying and specifying user requirements,
3. designing solutions, and
4. evaluating the outcomes of the design to assess its performance.

Iterations are repeated until the evaluation phase is satisfactory.

The term “user-centered method” was first used in 1986 by Don Norman (66), who argued the “*importance of design in our everyday lives, and the consequences of errors caused by bad designs*.” Ambler later highlighted the efficiency of agility (47) by demonstrating that UCD reduces computing costs (67). UCD approaches further provide advantages in a digital change context (68), all of which can be distinguished in four ways (69):

- User involvement increases the likelihood for a product to meet expectations which in turn increases sales and reduces customer services costs.
- Tailored products reduce the risk of human error (29, 70).
- Designer–user reconciliation increases empathy and creates ethical designs that respect privacy.
- By focusing on specific product users, designers recognize the diversity of cultures and human values through UCD—a step in the right direction to create sustainable businesses.

#### User-Centered Design in Digital Health

Digital health asserts a translational vision of changed practices and care systems [new modes of assessment through virtual reality (71) and the presence of sensors in smartphones for instance (27, 28)] to drive better health outcomes. However, the human–technology interaction was only put in light recently (72): it took a decade to first develop and then apply a theoretical understanding of the scope for a substantial, human-centered “design-reality” gap in healthcare (73).

In terms of functionalities, the focus is on usability of parameters such as appearance, appeal, and ease of navigation, as well as various interventions that include quizzes, games, self-monitoring tools, progress reports, downloadable documents, and other similar features [e.g., for social anxiety disorder (74)]. On the other hand, numerous barriers potentially prevent people from participating in evaluations of DHIs such as being too busy, feeling incapable of using the technology, or disliking its impersonal nature (75, 76).

Increasing interest in human factors has underpinned key developments in digital health, spanning intervention development, implementation, and the quest for patient-centered care (77). The emergence of ML chatbots and other patient-centered designs within Internet-based cognitive behavioral therapy has proven to facilitate access and improve tailored treatments (78). This is mainly due to the digital removal of several barriers such as reduced perceptions of stigma (very present in face-to-face services) and a rapid response to the need of “in the moment” support for mental distress. All these reasons increase the demand for digital mental healthcare in formal healthcare settings (79).

#### Benefits, Facilitators, and Barriers of UCD in DHI

To truly benefit from DHI, privacy and data governance, clinical safety (handling crisis in mental health apps for instance), and evidence for effectiveness must be at the core of the design (80, 81). This is unfortunately not always the case as shown by a smartphone app review revealing that, out of all health apps, only 11 were identified as “prescriptible” [meaning that they included randomized controlled trials (RCTs) reporting of effectiveness without clinical intervention] (82).

The UCD of digital health systems enables greater engagement and long-term use of digital tools (83). However, little attention is given to human factors such as ethnography of users or usability testing (77), or to the real-world difficulties that individuals face (84, 85) such as technology cost and privacy or security issues (86). These barriers reduce health outcomes with poor user engagement despite mobile health interventions (87–89). The decision-making power toward consumers is in turn insufficient (80), raising questions of access [namely in low- and middle-income countries (90)], equity, health literacy, privacy, and care continuity (14).

In their review of all barriers and facilitators for DHI engagement and recruitment, O'Connor et al. (91) distinguished four themes:

1. personal agency and motivation,
2. personal life and values,
3. the engagement and recruitment approach, and
4. DHI quality.

Education (91) and age (92, 93) were given particular attention as poor computer skills in both low-education individuals and old adults added to the enrollment struggle. In the same vein, literacy skills (94, 95) and the ability to pay for the technology (96) have impact on people's ability to interact with and use DHIs. All these factors ought to be further explored.

In summary, adopting a UCD of DHI would optimize long-term tool acceptance (6). Interdisciplinary collaborations could provide knowledge about “the context of use” (97), but it is crucial to further identify the technological and economic feasibility of the design (98, 99). In addition to the central role of human factors in DHI, attention should also be given to cognitive biases that come with ML strategy implementation and data interpretation.

### Identifying Cognitive Biases in Digital Health to Improve Health Outcomes

**Figure 3:**
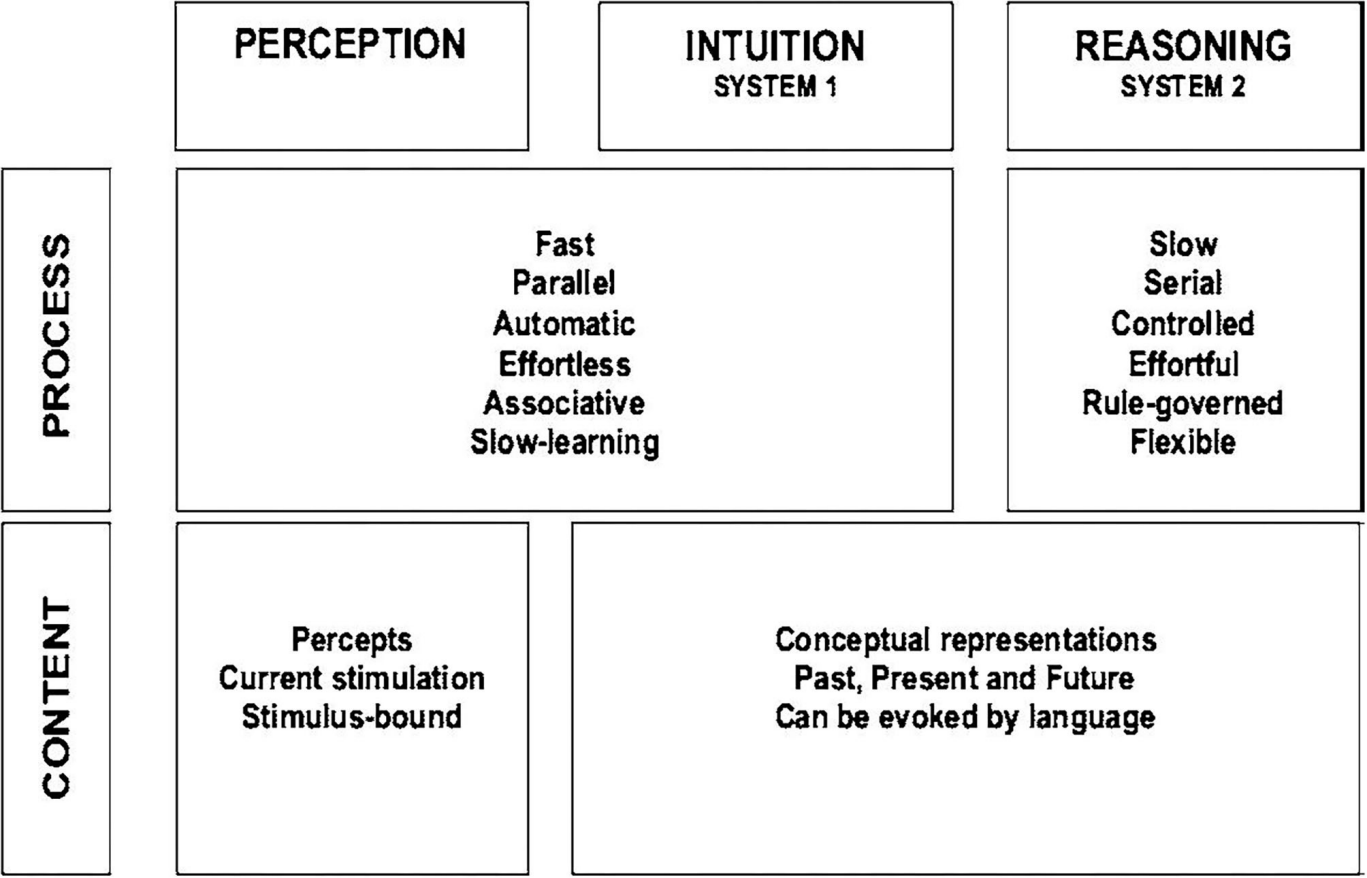
Properties of the dual-system theoretical framework of Kahneman (103) to explain judgments, decision under uncertainty, and cognitive biases: (2) system 1 refers to an automatic, intuitive, unconscious, fast, and effortless decision process; (3) system 2 makes deliberate, non-programmed, conscious, slow, and effortful decisions. Schema taken from Kahneman (103).

Studies from the past decades point at the vulnerability of the human mind to cognitive biases, logical fallacies, false assumptions, and other reasoning failures (100). In the health system context, cognitive biases can be defined as faulty beliefs that affect decision-making and can result in the use of heuristics in the diagnostic process (101, 102). Kahneman and Tversky introduced a dual-system theoretical framework to explain judgments, decision under uncertainty, and *cognitive biases* (103, 104). In this model, illustrated in Figure 3, system 1 refers to an automatic, intuitive, unconscious, fast, and effortless decision process. Conversely, system 2 makes deliberate, non-programmed, conscious, slow, and effortful decisions. Most cognitive biases are likely due to the overuse of system 1 vs. system 2 (100, 105–107).

#### Cognitive Biases Included in Diagnostic Reasoning and Healthcare Strategies

“*Diagnostic reasoning is the complex cognitive process used by clinicians to ascertain a correct diagnosis and therefore prescribe appropriate treatment for patients*” (108): the ultimate consequences of diagnostic errors include unnecessary hospitalizations, medication underuse and overuse, and wasted resources (109, 110).

Diagnostic reasoning and risk of errors can be explained by adapting the dual-system model to the health system context. For instance (99—see Appendix 2), system 2 overrides system 1 when physicians take a time-out to reflect on their thinking. System 1 also often irrationally overrides system 2 when physicians ignore evidence-based clinical decision rules that outperform them. Depending on what system overrides the other, the calibration (the degree to which the perceived and actual diagnostic accuracy corresponds) will differ.

The main cognitive biases affecting medical performance and diagnosis are the following (111, 112):

- *Premature closure* (113–117): an automatic process that occurs when the provider closes the diagnostic reasoning process by clinging to an early distractor/diagnosis without fully considering all the salient cues (106).
- *Search satisficing* (112, 118): a subtype of premature closure in which searches for further evidence are terminated after a diagnosis is reached. This is the case for medical students that do not initiate a search for a secondary diagnosis (118).
- *Availability* (106, 113, 118): falsely enhancing the probability of a diagnosis following the recent exposure of the physician to that diagnosis (106).
- *Anchoring* (112, 113, 115): a subtype of premature closure in which a provider stakes their claim on a diagnosis, minimizing information that do not support the diagnosis with which they have attached their proverbial anchor (115).
- *Base rate neglect* (119): predicting the diagnosis occurrence probability when two independent probabilities are erroneously combined, ignoring the base rate and leading to under- or overestimating the diagnosis possibility (120).
- *Diagnostic momentum* (112, 119, 121): a subtype of anchoring and premature closure in which the suggestion power of colleagues is taken at face value. For example, the diagnosis of anxiety disorder of the patient established from her family doctor through to the emergency department (ED), and although she might well have had hyperventilation due to anxiety, other possibilities were not ruled out earlier on in her care (112).
- *Overconfidence and lower tolerance to risk/ambiguity* (122, 123). Because of these two biases, misdiagnosis, mismanagement, and mistreatment are frequently associated with poorer outcomes, leading to patient dissatisfaction and medical complaints and eventually to a dropout of the digital health system (79, 124–126).

In the specific context of digital mental health, it is important to identify potential cognitive biases in patients as well in order to avoid misinterpretation and treatment misusage. In addition to the eight cognitive biases mentioned above, other cognitive factors such as coping strategies (127, 128) and the role of emotional stimuli (e.g., in depression, there is a lack of such a bias) (129) require particular attention in order to design tailored digital treatments and to drive ultimately an effective digital health strategy.

Early recognition of the cognitive biases of physicians is crucial to optimize medical decisions, prevent medical errors, provide realistic patient expectations, and decrease healthcare costs (107, 126, 130). Some debiasing strategies include the following:

1. Advocating for a view in which clinicians can change thinking patterns through awareness of bias and feedback (100). It consists of theories of reasoning and medical decision-making, bias inoculation, simulation training, computerized cognitive tutoring, metacognition, slow-down strategies, group decision strategy, and clinical decision support systems to force diagnostic reasoning out of bias-prone thought analytic processes.
2. Digital cognitive behavioral therapy [see, for review, 125] through which positive cognitive bias modification could be used as a potential treatment for depression (131), for anxiety disorders (25), for persecutory delusions (132), for improvement of social interaction in autism spectrum disorders and dementia (26), and for people with suicidal thoughts (133).

There is, however, no consensus regarding the efficacy of such debiasing approaches (118). In addition, other biases such as *aggregation bias* (the assumption that aggregated data from clinical guidelines do not apply to their patients) or *hindsight bias* (the tendency to view events as more predictable than they really are) also compromise a realistic clinical appraisal and could lead to medical errors (134, 135). This brings us to the urgent need for transparent and explicit data and strategy.

#### Biases in Defining Machine Learning Strategies

Cognitive biases exposed previously mainly concern physicians and their ability to analyze a digital diagnosis. Data scientists are also prone to specific cognitive biases given the strong interpretative component of data science and ML (136). Biases affecting data scientists in the digital mental health setting include but are not limited to the following (136):

- *survivorship*: a selection bias in which data scientists implicitly filter data based on some arbitrary criteria and then try to make sense out of it without realizing or acknowledging that they are working with incomplete data;
- *retrospective cost*: the tendency to make decisions based on how much of an investment they have already made, which leads to even more investment but no returns whatsoever;
- *illusion of causality*: the belief that there is a causal connection between two events that are unrelated;
- *availability*: the natural tendency to base decisions on information that is already available without looking at potentially useful alternatives that might be useful; and
- *confirmation*: the interpretation of new information in a way that makes it compatible with prior beliefs.

Despite these data science biases, a promise of ML in healthcare is precisely to avoid biases. The biases of scientists and clinicians would be circumvented by an algorithm that would objectively synthesize and interpret the data in the medical record and/or offer clinical decision support to guide diagnosis and treatment (58). In the digital health context, integration of ML to clinical decision support tools such as computerized alerts or diagnostic support could offer targeted and timely information that would in turn improve clinical decisions (58, 137–140). With the rise of ML in the DHI, data sources and data collection methods should be further examined to better understand their potential impact (141–143). Biases that could be introduced through reliance on data derived from the electronic health record include but are not limited to the following:

- *Missing data*: If communicated sources such as patient-reported data are incomplete (missing or inaccessible), algorithms (that only use available data) may correctly misinterpret available data (144). Algorithms could thus be a bad choice for people with missing data (145) [people with low socioeconomic status (146) or those with psychosocial issues (147) for instance].
- *Misclassification and measurement errors*: Misclassification of diseases and measurement errors are common sources of bias in observational studies and analyses based on electronic health record data. Care quality may be affected by implicit biases related to patient factors, such as sex and race, or practitioner factors [e.g., patient with low socioeconomic status (148) or women (149)]. If patients receive differential care or are differentially incorrectly diagnosed based on sociodemographic factors, algorithms may reflect practitioner biases and misclassify patients based on those factors (58).

We mostly identified and described biases that interfere once the data are already collected. It is important to note that biases can also interfere earlier in the process, at every step of it, from brainstorming to literature reviewing (143). The main recommendation is to stay alert to all different biases, whether they are mentioned in this paper or not.

## Data Collection–Analysis

### From Data to Information

As seen above, decision-making in the medical field often has far-reaching consequences. To better measure these consequences, it is essential to build certainties: certainties on the data used, their source, their format, and their update; certainties on the information put forward and their implications; and certainties on the tools exploiting these data as well as on the reliability of the algorithms and visual representations made available. These questions concern data in a broad way.

It thus seems important to start this technical part of the paper by defining the notions of data, information, and knowledge, as all three are involved in decision-making processes.

We will then focus our approach on the data and the different steps to structure, exploit, and enhance them.

#### Definitions: Data, Information, and Knowledge

Grazzini and Pantisano (150) defined each concept as follows:

*Data* can be considered as raw material given as input to an algorithm. Since it cannot be reproduced when lost, it must be carefully preserved and harvested. It can be of different forms: a continuous signal as in the recording of an electroencephalogram (EEG), an image representing a magnetic resonance imaging (MRI), a textual data, or a sequence of numerical values representing a series of physiological measurements or decisions taken *via* an application. Data can be complete, partial, or noisy. For example, if only a portion of an EEG recording is available, then the data are partial. Conversely, an EEG recording that has been completed but that has some parts unusable is said to be noisy because the noise alters the completeness of the recording. Two types of data can be distinguished: unstructured data, i.e., data directly after their collection or generation, and structured data, i.e., data that have been analyzed, worked on, and put in relation to each other to put them in a format suitable for the analysis considered afterwards. In the second case, it is considered information. Importantly, data by themselves are worthless.

*Information* is dependent on the original data and the context. If it is lost, it can be reproduced by analyzing the data. Depending on the data processed at time *t*, information must be accurate, relevant, complete, and available. Information is intelligible by a human operator and can be used in a decision-making process. It is therefore significant and valuable since it provides an answer to a question. It can take various forms such as a text message, a table of numerical values, graphs of all kinds, or even in the shape of a sound signal.

When semantics are added to a set of information, it becomes *knowledge*. Information, depending on the context, will not have the same impact. It is the context and the semantics brought by it and the human operator involved that will determine the value of that knowledge.

To illustrate these definitions in a mental health setting, in the case of a patient undergoing a follow-up with a psychiatrist: the psychiatrist can make his patient pass numerous tests in order to collect data: MRI, EEG, and textual answers to questionnaires. These data, once processed, formatted, and analyzed together, will represent a set of information on the condition of the patient. It is the combination of the knowledge and experience of the doctor, combined with his knowledge of the patient, his family context, and the current socioeconomic context, that will enable him to have a global knowledge of his patient and to provide him with the best possible support.

**Figure 4:**
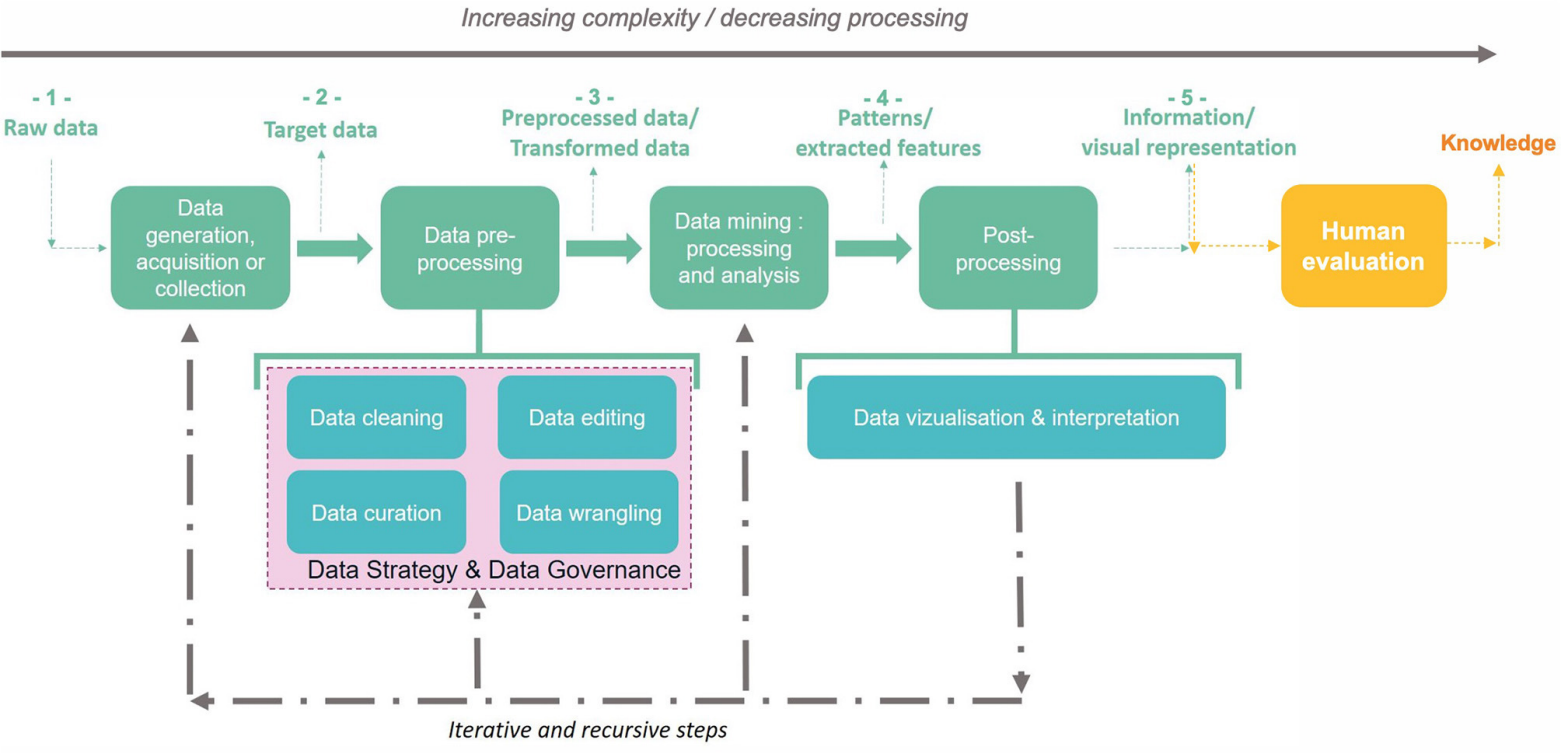
Data transformation into information (from 1 to 5) then knowledge through KDD steps: (a) raw data can be collected to become target data, which can also be acquired or generated by an external process: it is the data generation, acquisition, or collection step. (b) Target data are submitted to a data preprocessing step according to the data mining techniques targeted: target data can thus be cleaned, filtered, completed, and anonymized if necessary. This second step allows to obtain preprocessed data. (c) Preprocessed data are submitted to data mining techniques to detect, identify, and extract patterns and relevant features. (d) Discovered patterns and features can thus be rearranged (e.g., with visual tools) for or within the phase of interpretation during the postprocessing step. The result is the generation of information. (e) Information evaluated by a human becomes knowledge: this step is an external one (i.e., not technical) and takes into account knowledge of the situation (context, issues, stakeholders, etc.).

The passage from data to information thus requires a majority of digital processing to highlight correlations according to a given context. However, the passage to the knowledge stage requires considering individuals involved (see *Project design*). Figure 4 illustrates data transformation into information through digital processing and into knowledge through human evaluation.

There is thus an increasing complexity in this process of transforming data into information and then into knowledge which make it difficult to identify and extract. We will present a process dedicated to these tasks in the following section.

#### Knowledge Data Extraction in the Literature

The process of Knowledge Discovery of Data (KDD) is defined as the process of discovering useful knowledge from data (151). As a three-step process, the KDD includes (2) a preprocessing step which consists of data preparation and selection, (3) a data mining step involving the application of one or many algorithms in order to extract information (i.e., patterns), and (4) a postprocessing step to analyze extracted information manually by a human operator and lead to knowledge discovery.

As an iterative and interactive process, KDD involves many steps and decisions of the users. Iterations can continue as long as extracted information does not satisfy the decision-maker (see *Identifying cognitive biases in digital health to improve health outcomes*).

**Figure 5:**
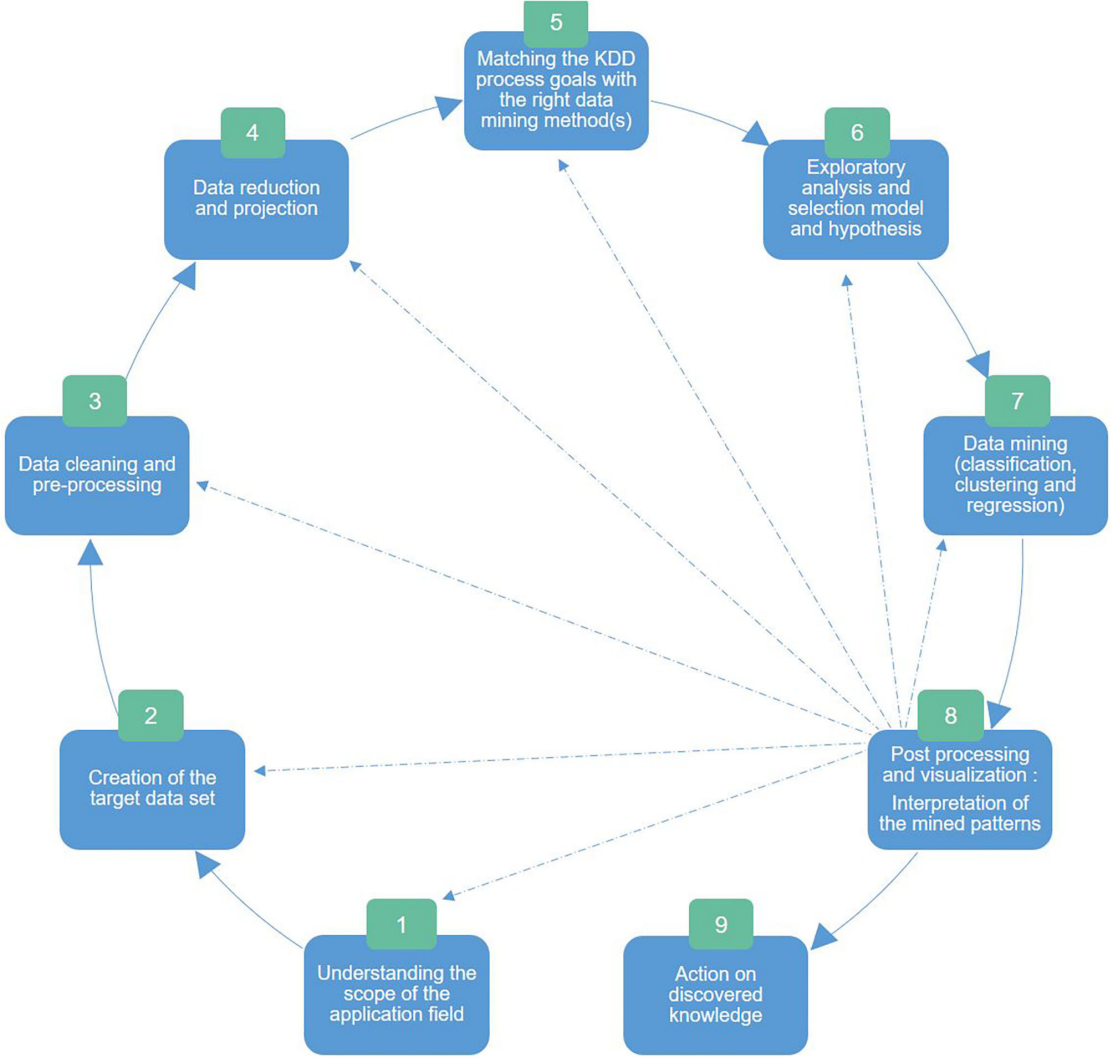
The nine Knowledge Discovery of Data stages adapted from Fayyad (151): (2) understanding the scope of the application field, (3) creation of the target dataset, (4) data cleaning and preprocessing, (5) data reduction and projection: dimensionality reduction and extraction of invariant representations and relevant characteristics, (6) matching the KDD goals with the right data mining method(s), (7) exploratory analysis and selection model and hypothesis: selection of the data mining algorithm and method that will be used for the pattern search, (8) data mining: searching for interesting patterns in a particular form of representation (e.g., tree classification, regression, and clustering), (9) data postprocessing and visualization: interpretation of the patterns found with possible return to any step from 1 to 7 for a new cycle, and (10) action on discovered knowledge.

Concretely, as illustrated in Figure 5, the KDD stages encompass the following: (2) understanding the scope of the application field; (3) creation of the target dataset; (4) data cleaning and preprocessing; (5) data reduction and projection: reducing the number of variables to be analyzed by reducing the dimensionality of the data, extracting invariant representations, or searching for relevant characteristics; (6) matching the goals of the KDD process with the right method(s) in data mining; (7) exploratory analysis and selection model and hypothesis: selection of the data mining algorithm and method that will be used for the pattern search; (8) data mining: searching for interesting patterns in a particular form of representation, which includes rule and tree classification, regression, and clustering; (9) data postprocessing and visualization: interpretation of the patterns found with possible return to any step from 1 to 7 for a new cycle; and (10) action on discovered knowledge.

Here, we mainly focus our approach on the technical aspect, i.e., data and their transformation into information. We aim to present a complete and global approach by covering the KDD stages in the life cycle of a digital health product from the definition of the scientific question to data collection and analysis (for further details, see Appendix 3). We will reveal our approach in the following section.

#### Information Data Extraction Applied to Technology

We are aligned with the three-step approach of Fayyad (151) for information extraction:

- data preprocessing (data cleaning, data editing, data curation, and data wrangling),
- data mining with a special focus on biostatistics and AI and ML algorithms, and
- postprocessing focusing on data visualization.

Figure 4 proposes a representation of the global approach for information extraction for a specific question or product design.

Upstream of these activities, we would like to highlight two areas that are essential to good data management and that allow an optimization of the research of a team: data strategy, which aims at standardizing data management, and data governance, or the implementation of solutions to respond to the strategic issues defined beforehand.

### Global Approach: Data Strategy and Governance

#### Data Strategy

Within a digital research project, technical and operational tasks are either managed by the same person (it is generally the case in a fundamental research team) or by distinct groups (it is the case for R&D groups in which technical teams focus on system architecture, development, quality, and testing, while operational teams handle experimental requirements and process definition). These concepts are classically and poorly applied to data (in fundamental and R&D teams), thus slowing down the improvement of accuracy, access, sharing, and reuse of data (152).

Data strategy applied to research aims at using, sharing, and moving data resources efficiently (adapted from 147) in order to manage projects easily, facilitate scientific collaboration, and accelerate decision-making regarding new project ideas.

Data strategy contains five core components that work together to comprehensively support an optimal data management:

- *Identification*: to set common data definition shared with the team, collaborators, and more broadly with the scientific community. In the mental health context for instance, it is crucial to define all biomarkers (whether they are genetic, molecular, anatomical, or environmental) to encompass the complexity of psychiatric disorders.
- *Storage*: to maintain data in information technology (IT) systems that allow easy access, sharing, and data processing.
- *Provision*: to anticipate and to prepare data in order to directly share or reuse it with adequate documentation explaining rules and definition.
- *Processing*: to aggregate data from different IT systems and obtain a centralized 360° data vision. In a mental health project, it could be pertinent to aggregate clinical, biological, and imaging data for instance.
- *Governance*: see dedicated chapter below (*Data governance*).

#### Data Governance

According to the Data Governance Institute, data governance is “*a system of decision rights and accountabilities for information-related processes, executed according to agreed-upon models which describe who can take what actions with what information, and when, under what circumstances, using what methods*.” Data governance is generally informal in fundamental research labs due to reduced (<15 people) and homogeneous (with the same background) teams in which processes, information, and tools are shared by everyone. This informal approach is less applicable with team expansion, different profile recruitment, scientific collaboration, or any operation that implies cross-functional activities. Initially designed for private industries, formal data governance approaches allow to frame cross-functional activities with a set of objectives adapted from the Data Governance Institute:

- to optimize decision-making: with a deeper knowledge of data assets and related documentation. This is helpful for instance when a choice has to be made between several scientific projects or strategies and the formal data governance approach estimates the ratio between investment and expected scientific value;
- to reduce operational friction: with defined and transparent roles and accountabilities regarding data and data use;
- to protect the needs of teams within a scientific collaboration framework;
- to train teams and collaborators to build common standards for approaching data issues;
- to reduce costs and increase effectiveness through effort coordination;
- to ensure transparency of processes;
- to accelerate and facilitate scientific collaboration;
- to allow scientific audit; and
- to respect compliance with the required documentation.

Data governance should not be applied as a theoretical concept but should rather be considered for its potential added value when it comes to pain points and the definition of use cases (e.g., to ensure data quality of a mental health digital project focused on schizophrenia). Good practices could anticipate value creation and changes triggered by the data governance framework (harmonious collaborations and their impact on data-related decisions for instance).

Regarding enterprise systems, research teams and/or scientific collaboration will require only a restricted number of rules and thus do not need a large organization assigned to data governance but more likely some clear and identified accountabilities and documentation for all scientific members (for further details, see Appendix 4).

In summary, data strategy and governance give a starting framework to structure the data management policy and strategy of a research team. Depending on the size of the team, the issues at stake, and the collaborations, these steps can have a real added value. As research teams do not rely on nor need large organizations for their data governance, it is also important to include other operational steps in a digital health data-centered project.

### Operational Approach: From Preparation and Mining to Visualization

#### Data Preprocessing: Cleaning and Making Data Available

The preprocessing step consists in preparing the dataset to be mined (see Figure 4). This implies the following: (2) *data cleaning*, which consists in removing noise, corrupted data, and inaccurate records (3, 153, 154) *data editing* to control data quality by reviewing and adjusting it (155) and to anonymize data when needed with respect to data privacy standards (4, 156, 157) *data curation* to manage data maintainability over time for reuse and preservation (158); and 4) *data wrangling* or the process of mapping data from one type to another to fit the selected mining technique (e.g., from natural language to numerical vectors) (159). It is an important step in the KDD process (160) since the quality of the analysis of a data mining algorithm relies on the data available for the analysis. This step is inevitable as each dataset must be preprocessed before being mined. Alternatives (161) to preprocessing data exist but depend on the objective and the nature of available data, which makes it overwhelming to unexperienced users (162). It is thus essential to fix an explicit objective (i.e., a question to answer or a hypothesis to study) before preprocessing to choose the appropriate techniques.

#### Data Mining: From Biostatistics to Machine Learning

##### Biostatistics

Unlike ML, biostatistics are not used to establish predictions; hence, they do not require a large amount of data. Biostatistics study inferences between different populations by establishing a quantitative measure of confidence on a given sample of the population (163).

The frontiers between statistics and ML can be blurry as data analyses are often common to both [it is the case for the bootstrap method used for statistical inference and for the random forest (RF) algorithm]. It is thus important to differentiate statistics (that require us to choose a model incorporating our knowledge of the system) from ML (that requires us to choose a predictive algorithm by relying on its empirical capabilities) (163).

##### Data Mining

Data mining is characterized by the willingness to find any possible means in order to be able to answer the research question. It can thus be defined as the process of analyzing large amounts of data to uncover patterns, associations, anomalies, commonalities, and statically significant structures in data (164). The two main goals of *data mining* are thus *prediction* of future behavior according to discovered patterns and *description* or the presentation in human-understandable shapes of the patterns found. To do so, data mining focuses on the analysis and extraction of features (extractable measurements or attributes) and patterns (arrangements or ordering with an underlying structure). Subfields of data mining include pattern recognition domain (or the characterization of patterns) (165) and *pattern detection* and *matching* (mining data to characterize patterns).

Data mining also includes subsets of popular algorithms:

- *Classification* consists in learning a function that classifies data into one or more predefined classes. For example, to predict generalized anxiety disorder among women, it is possible to either use RF to implement featured selection of the data mining classifier on the mental health data (166), or to use decision tree-based classification (167) or Shapley value algorithm (168).
- *Regression* consists in learning a function that matches data with a real predictor variable. The purpose of these algorithms is to analyze the relationship of variables with respect to the others, one by one, and to make predictions according to these relationships. It can be a statistical method or a ML algorithm. For example, Yengil et al. (169) used regression algorithms to study depression and anxiety in patients with beta thalassemia major and to further evaluate the impact of the disorder on quality of life.
- Another type of algorithm is *clustering* that consists in detecting a finite set of categories to describe data. Categories can be mutually exclusive and exhaustive or consist of a richer representation such as hierarchical or overlapping categories. The k-mean algorithm for instance can describe a population of patients as a finite set of clusters, each one grouping individuals sharing same features (e.g., children vs. adults).
- *Summarization* methods are used to find a compact description for a subset of data.
- *Dependency modeling* consists in finding a model that describes significant dependencies between variables. This can be done at the structural level (specifying dependent variables) or the quantitative level (specifying the strength of a dependency using numerical scales).

All these methods aim at extracting features or patterns following the search method as previously discussed in *Defining the goal and the approach*.

#### Data Postprocessing: Visualization and Evaluation

To efficiently communicate scientific information, data visualization (or graphic representation) should be specifically designed for the targeted audience. This can involve exploratory and/or explanatory objectives (170):

- *Pure exploratory*: addressed to teammates and collaborators to highlight main results in order to make data memorable and to identify the next strategic steps of the project.
- *Explanatory/exploratory mixed*: addressed to the scientific community, to share information and provide reliable (accessible and intelligible) data that can be analyzed and challenged by others. It can also support the scientific story telling in a grant application.
- *Pure explanatory*: addressed to patients, to quickly and efficiently explain scientific information with an appropriate and tailored content.

As seen in Figures 4, 5, evaluating and interpreting mined patterns or extracted data through visualization can possibly induce returning to any previous step from preprocessing to data mining until discovered knowledge answers the fixed goal.

### An Example of Our Method Applied to Mental Health

There is a growing number of mobile apps dedicated to mental health. Among them, “Moodfit” shapes up the mood, “Mood mission” teaches coping skills, “Talkspace” provides a virtual space for therapy, “Sanvello” acts as a stress relief, “Headspace” opens a virtual door to meditation, and “Shine” answers the specific mental health needs of BIPOC communities. However, there is no single guide for the development of evidence-based MHapps (171). An analysis of all apps dedicated to depression on the major marketplaces (Apple App and Google Play stores) shortlisted 293 apps that self-advertised as research-based (172). Among these apps, only 3.41% had published research that supports their claims of effectiveness, among which 20.48% were affiliated with an academic institution or medical facility. This analysis strongly indicates the need for mental health applications to be more rigorous (172), i.e., by following a strict method.

We have thus applied our end-to-end methodology to build a mobile application called i-decide (www.i-decide.fr) that facilitates decision processes under uncertainty. The application aims at complementing existing neuropsychological testing that take places punctually in a controlled setting by collecting longitudinal data on a daily basis. The data collected concern decision processes and all cognitive and emotional functions that impact decision-making (Boulos et al., in revision). All data are used to feed an algorithm that learns optimal choices (that reduce long hesitations and associated anxiety as well as the percentage of regret postdecision) under uncertain conditions. We tested the application on a population of 200 adult users with no diagnosed mental illness. Results revealed time slots during which decision-making was optimal as well as clusters of decision profiles according to stress, motivation, daily goals, support system, and the ratio of minor vs. major decisions (Boulos et al., in revision). More information can be found on the mobile application's website www.i-decide.fr.

## Discussion

### Summary

AI algorithms together with advances in data storage have recently made it possible to better characterize, predict, prevent, and eventually cure a range of psychiatric illnesses. Amid the rapidly growing number of biological devices and the exponential accumulation of data in the mental health sector, the upcoming years are facing a need to homogenize research and development processes in academia as well as in the private sector and to centralize data into federalizing platforms. In this work, we describe an end-to-end methodology that optimizes and homogenizes digital biophysiological and behavioral monitoring with the ultimate ambition to bridge the gap between fundamental and applied research.

### Methodology and Recommendations

The first step described project conception and planning stages. We proposed approaches to evaluate the feasibility of a digital mental health project, to define its goal, and to design the research approach accordingly. We clarified digital mental health research conceptions and misconceptions and described the difficulties of combining academic literature and market research. We further underlined the importance of collaborations in the interdisciplinary and intersectoral field to better understand what digital mental health is. We finally focused on the concrete planning of such methodology, that is, how to inject agility every step of the way to create ultimately platforms that reconcile different stakeholders to provide the best assistance possible to patients with mental health issues.

The second step zoomed in on the specificities of project design in mental health. We explained the importance of digital health interventions, the necessity to have clear goals, and the importance of human factors in defining them (introducing the user-centered design). We finally described cognitive biases and their impact on both physicians and data scientists in digital mental health.

The third, last, and more technical step described the stages from data collection to data analysis and visualization. We differentiated the notions of data (raw element), information (transformed data), and knowledge (transformed data with semantic contextual value) to then focus on the key steps of data in a digital mental health research. We provided recommendations for data management, strategy, and governance depending on the size and type of research structure and further elaborated a KDD-based operational approach that can be especially useful for small research teams that wish to work from collection to processing.

### Issues at Stake: Ethics and Biases

Exploring the literature around digital mental health interventions leads us to question existing practices, that is, both their strengths and their issues. There are so many questions the scientific community and other stakeholders should consider when developing digital mental health solutions, and these include ethics and biases.

For a trial to be ethical, the assumption of equipoise (i.e., equilibrium) should be included in the design. While general designing and conducting RCT principles (173) are applicable to DHIs, specific DHI features deserve consideration when a trial is expected to provide evidence for rational decision-making: (2) the trial context, (3) the trade-off between external validity (the extent to which the results apply to a definable group of patients in a particular setting) and internal validity (how the design and conduct of the trial minimizes potential for bias) (174, 175) (e.g., of poor trade-off: recruiting highly motivated participants because of missing follow-up data) (174), (4) the specification of the intervention and delivery platform, (5) the choice of the comparator, and (6) establishing methods for separate data collection from the DHI itself.

Detailed specification of DHI is important, because it is required for the replication of trial results, the comparison between DHIs, and synthesizing data across trials in systematic reviews and meta-analyses (176). The relevant data to collect would then focus on usage, adherence, demographic access parameters, and user preferences (6, 177), even if participants are biased because they have access to a myriad of other DHIs. Indeed, someone who has sought help for a problem, entered a trial, and been randomized to the comparator arm, only to find the intervention unhelpful, may well search online until they find a better resource (178).

Finally, a well-designed RCT, especially for its ethical part, highlights the need to create interdisciplinarity. Researchers in digital mental health could learn from the multicycled iterative approach adopted in the industry for optimized development. Researchers from an engineering or computer science background may be surprised by the reliance on RCTs, whereas those from a biomedical or behavioral sciences background may consider that there is too much emphasis on methods other than RCTs. By enhancing critical thinking, interdisciplinarity in a team also tends to reduce cognitive biases. Although we have dedicated an entire part of this paper to cognitive biases (*Identifying cognitive biases in digital health to improve health outcomes*), there are several important points yet to be discussed. This includes the impact of biases in the decision-making process in digital mental health, the repercussion of the biases of practitioners on the data, and the biases of algorithms. One important message is that there are numerous cognitive biases across multiple domains (such as perception, statistics, logic, causality, social relations…) and that these biases are generally unconscious and effortless, making them hardly detectable and even less so controllable (179). Another important point is how AI and ML acceptability by the community on a social level can in turn affect the cognitive biases of physicians, researchers, and patients on digital mental health. In Appendix 5, we discuss these different issues and propose recommendations to better control the impact of cognitive biases in digital mental health research with the ultimate ambition to improve diagnostic reasoning and health outcomes.

### Technical Challenges

In addition to the ethical considerations, working with data comes with technical challenges, three of which we wish to highlight: (2) interoperability that is defined as the property that facilitates rapid and unrestricted sharing and use of data or resources between disparate systems *via* networks (180), (3) the trade-off between anonymization (to respect data privacy standards) and anonymization willingness, and (4). ML interpretability and explainability issues in digital mental health and digital health in general.

The multiplicity of tools that needs to be functional all while operating easily with other tools rushed the need for a “plug-and-play” interoperability. This is particularly the case for the medical field and its daily clinical use of various medical devices (MRI, computed tomography, ultrasound…). Beyond the traditional interoperability between different healthcare infrastructures, the will of patients to consult and understand their own data is imposing a new infrastructure-to-individual interoperability (181). In the light of this context, we believe that interoperability should be considered by a research team for their data strategy, especially when the research involves collaborations that are wanted or already in place. Beyond optimizing the collaboration and facilitating patient contribution, this could avoid data manipulation mistakes, as well as security or confidentiality failure.

Beyond confidentiality, one of the most sensitive points is privacy. In the context of digital mental health, and given the fact that it is a relatively young field with little information regarding clinically relevant variables (157), the bigger the data volume, the easier it is to identify relevant variables. The need for large data volumes is, however, challenged by the difficulty to collect these data all while respecting the strict health ethics and laws. It is thus crucial to set up the right privacy strategy. We would like also to highlight the existence of other technical challenges such as anonymization of data and explainable AI that are growing research fields (for further details, see Appendix 6).

## Conclusion

In conclusion, our interdisciplinary collaboration to provide an end-to-end methodology for digital mental health research using interpretable techniques and a human-centered design with a special attention to data management and respecting privacy is therefore (2) a moral subject because it is linked to the transparency of the algorithms and, by extension, to the deriving decisions; (3) an ethical subject because it requires taking into account all people involved, their cognitive biases, and their impact on trials, experiments, and algorithms; and (4) a lever of trust for the end user specially in the mental health field where personal privacy is a critical but essential part that has to be respected.

Beyond this work, we find through our review of the literature that the various approaches taken to address different facets of product conception and design from research to market are siloed. Advances are often made separately and little attention is given to interdisciplinary and intersectoral centralizing approaches like ours in an attempt to provide a complete end-to-end methodology. We cannot stress enough on the timely importance of collaborations in digital mental health to reduce the disciplinary and sectoral gap and create platforms that deliver solutions trusted both by scientists and end users.

## Data Availability Statement

The original contributions presented in the study are included in the article/Supplementary Material, further inquiries can be directed to the corresponding author/s.

## Author Contributions

All authors listed have made substantial, direct and intellectual contribution to the work and approved it for publication.

## Funding

We express our gratitude to onepoint, especially Erwan Le Bronec, for the financial support to the R&D department, which permitted us to carry out this work. The funder was not involved in the study design, collection, analysis, interpretation of data, the writing of this article or the decision to submit it for publication. The only contribution of the funder is to pay the preliminary publishing fees.

## Conflict of Interest

AD, AM, and ICK were employed by the company onepoint when the research was conducted. The remaining author declares that the research was conducted in the absence of any commercial or financial relationships that could be construed as a potential conflict of interest.

## Publisher's Note

All claims expressed in this article are solely those of the authors and do not necessarily represent those of their affiliated organizations, or those of the publisher, the editors and the reviewers. Any product that may be evaluated in this article, or claim that may be made by its manufacturer, is not guaranteed or endorsed by the publisher.
