# Supplementary material for: An Iterative and Collaborative End-to-End Methodology Applied to Digital Mental Health
Source: Front Psychiatry. 2021 Sep 23;12:574440. doi: 10.3389/fpsyt.2021.574440 (PMC8495427; doi:10.3389/fpsyt.2021.574440)
Supplement: Supplementary file 1 [file Data_Sheet_1.docx]

Title

Appendix : An iterative and collaborative end-to-end methodology applied to digital mental health

Boulos LJ^1^, Mendes A^2^, Delmas A^2^, Kaadoud IC^2^

1: Saint-Joseph University, Beirut, Lebanon

2: Groupe onepoint, Paris, France

Appendix.1 Reviewing the literature

As a research project is built on the foundations of previously accumulated knowledge, a thorough understanding of the topic to be researched and its status quo should be carried out before the start of the study (Eston & Rowlands, 2000). This allows at least to verify that the research project has not already been conducted by someone else. In the case of digital health technologies, this also allows to see what has already been substantiated (Mathews et al., 2019) and to estimate whether the project’s outcome will still bring additional value by the time the packaged solution reaches the market (Loncar-Turukalo et al., 2019). Such literature can also indicate the rate at which research outcomes are delivered, and thus the rate at which reviewing the literature should be iteratively made in the process of the research itself.

Reviewing the literature is the most exploratory aspect of research (Taylor & Pagliari, 2018). It thus has a veryhas very unclear methodology leaving room for several biases in the selection of articles (Eston & Rowlands, 2000). Most literature searches begin with one of the electronic databases available for the given field (PubMed, The Cochrane Library, Web of Science, Elsevier, and ProQuest), using a combination of carefully selected keywords. In the context of digital health, sources of information are widely scattered thus harder to gather and synthesize (Guise et al., 2014). Publications are found both in biomedical and healthcare databases, as well as in engineering literature (Deshazo et al., 2009). These databases are limited as to which journals are listed. To avoid missing pertinent articles, it is advised to search more than one electronic database (Loncar-Turukalo et al., 2019). Further relevant papers may be obtained by studying the reference lists of “useful” on topic articles. The challenge in digital health however lies more in the selection of articles: so much is available, how decide what is appropriate and what is not (Taylor & Pagliari, 2018)? As it is almost impossible to obtain all papers addressing one research question, researchers often find themselves narrowing the search strategy. The selection of papers should be carefully made as it necessarily induces biases (Taylor & Pagliari, 2018) (see Part 3.2). Limiting a topic can be done by geographical area, by language, by time frame, by discipline, by population group. Either way, considerations when selecting articles should include i) different points of view, ii) more elusive papers, iii) a random selection of relevant papers, and iv) papers that are hard to find. In addition, considerations when determining the scope of a research topic include time, money interest and commitment, as well as impact to self and others.

Appendix.2 A model for diagnostic reasoning from Ely et al, 2011


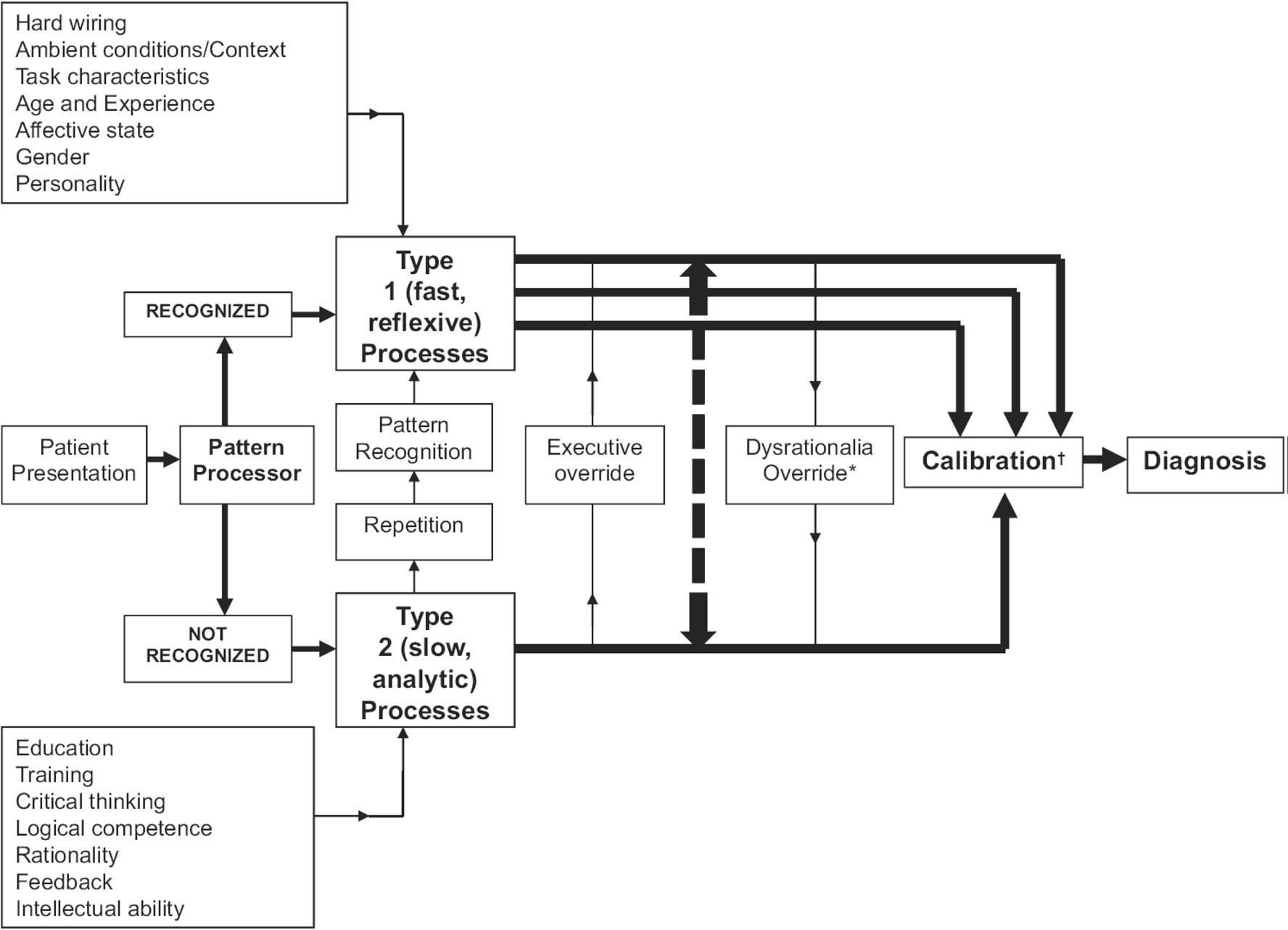


A model for diagnostic reasoning from (Ely et al, 2011) based on Figure 3 : System 2 can override system 1 when physicians take a time-out to reflect on their thinking. System 1 overrides system 2 when physicians ignore evidence-based clinical decision rules that outperform them.

Appendix.3 Additional recommendations for DHI design

Beyond the KDD steps described in section 4.1.2 , studies suggest specific recommendations for data management and usability when designing DHI.

One set of recommendations (among them, Murray and colleagues (2016)) can be useful to generate more data as the lack of data in the health field often represents an obstacle to analysis: 1) considering and validating appropriate short-term proxy outcomes; 2) improving methods for early formative work and defining whether further investment in more intensive research designs is needed; 3) better understanding of how to improve internal validity of DHIs in RCTs (in terms of retention and follow-up) without jeopardizing external validity (in terms of the recruited population or impact on the intervention); 4) improved methods for reducing the large amounts of missing data that may occur, and addressing the inevitable biases this raises; and 5) better methods for determining whether and how a DHI will become scalable and sustainable, including understanding how a DHI might be supported through self-sustaining business models.

Another set of recommendations can help improve comparisons of data generated by different studies (Murray et al., 2016): 1) identification, specification, and classification of important contextual factors; 2) specification and classification of target populations; 3) specification and classification of DHIs, to gain an understanding of the important active components and mechanism of action, to replicate and synthesize evidence across DHI evaluations, and begin to address the issue of determining substantial equivalence between DHIs; 4) specification and determination of appropriate comparators, according to the stage of the research process; and 5) improved reporting of studies of DHIs, building on initiatives such as the Template for Intervention Description and Replication reporting guideline (Hoffmann et al., 2014) or the CONSORT_EHEALTH statement (Eysenbach & CONSORT-EHEALTH Group, 2011).

In addition to these data-related recommendations, other recommendations (Huckvale et al., 2015; Murray et al., 2016) address the need to include harm evaluation that is, the harmful potential of a research project (breaches of privacy for instance). More broadly, there is a need for educational communication around the risks and regulations of DHI. This would protect both users and researchers all while reducing DHI resistance due to lack of information.

Appendix.4 Data Governance accountabilities and documentation

Formal Data Governance set rules rely on accountabilities and documentation:

- **Data dictionary**; centralized metadata to identify and characterize data (e.g. label, definition, format, owner, users, source...) (Bourne, 2014). With the exponentially growing amount of scientific data in digital mental health (Bucci et al., 2019), a dictionary allows to maintain and manage metadata thus supporting “by design” data curation.
- **Data Governance policies;** defined data-related roles and accountabilities (such as data owners and/or users) as well as data accesses and processes.
- **Quality requirements;** establishment of the minimum quality level required to analyze data (Sebastian-Coleman, 2012). This level is defined by the data owner based on pre-defined criteria: wholeness (empty fields are not allowed), validity (data format is respected), integrity (the data is raw), coherence (the data is coherent either with a specific context or with other data), etc.
- **Control lists;** based on quality requirements, manual and automatic controls prevent scientific misinterpretation by measuring data quality and usability. They also require transparent processes as well as accountable and exhaustive documentation. In the case of health apps, it is generally impossible to save information when the form is incomplete (because of critical data lacking). In the absence of Data Governance, researchers use manual quality controls. It is the case for too noisy EEGs that are analyzed manually to discard low quality data.
- **Data lineage;** describes the data lifecycle including where it is generated (data generation, acquisition or collection), where it is stored, where it is transformed (data pre-processing) and where it is consumed (data analysis). If data quality decreases, this document is crucial to target IT systems in order to monitor data quality in each of them, then to identify action allowing data quality improvement.

Regarding enterprise systems, research teams and/or scientific collaboration will require only a restricted number of rules and thus do not need large organization assigned to Data Governance but more likely some clear and identified accountabilities for all scientific members.

In summary, Data Strategy and Governance give a starting framework to structure the data management policy and strategy of a research team. Depending on the size of the team, the issues at stake and the collaborations, these steps can have a real added value. As research teams do not rely on nor need large organizations for their Data Governance, it is also important to include other operational steps in a digital health data-centered project.

Appendix.5 Issues at stake

Impact of biases on the decision-making process

The design of a Digital Health Interventions (DHI) involve many persons: the designers, the developers, the testers, the humans being, the data (collection or generation) and finally the users whether they are individual looking for a wellbeing app, or physicians looking for an assistant tool for decision making. More broadly, cognitive biases impact all the steps of the end-to-end methodology described in the paper which includes human being, from the project idea to the data post-processing.

Despite protocols, established process, and explicit rules, cognitive biases are present in each one of the individuals that intervene in the design of the DHI. There are many studies of cognitive biases in numerous works in cognitive psychology, social psychology and more generally in the cognitive sciences (Elstein, 1976, 1983; Marewski & Gigerenzer, 2012; Tversky & Kahneman, 1974; Wegwarth et al., 2012). Among their results, two important points: 1) there are numerous cognitive biases specific to the human mind across multiple domains like perception, statistics, logic, causality, social relations, etc., 2) these cognitive biases are generally unconscious and effortless which make them more difficult to detect, to prevent or even correct, and that lead, among others, to errors of perception, evaluation and logical interpretation.

Their characterization is important both in the judicial and scientific fields since they are harmful in a logical process, so that is why we choose to make a special focus on the impact of the cognitive bias in the research field of mental health from data to algorithms.

Practitioner biases impact on data

Physicians, practitioners and clinicians, as all individuals, have also cognitive biases that impact their reasoning process and behavior. We thus think that this socio-professional category, whose decisions can have a heavy impact on peoples’ health, should take the initiative to self-monitor specially when more analytic thought is necessary to avoid errors of perception, evaluation or interpretation.

An improved awareness may pass by increasing knowledge, reflecting reasoning and expertise and, the most important, getting help from colleagues exerts and tools, such as DHI (Croskerry, 2013). To help prevent certain errors in charged situations with a lot of uncertainty, systematic solutions have also been proposed, such as computerized decision support or others. For example, Schiff & Bates (Schiff et al., 2010) put in light the interest to improve electronic documentation to avoid diagnostic errors. In the same vein, literature shows that decision rules and algorithms will outperform or match the performance of the physician about 90% of the time (Saposnik et al., 2016). However, the uptake of clinical decision rules is abysmally low because of overconfidence bias of the physicians who think – wrongly – they can outperform computers and that algorithms and clinical decision rules decrease their autonomy (Saposnik et al., 2016). To counter this and in the context of CoVid-19, where the degree of uncertainty leads to maximize our cognitive bias, one way which can facilitate acceptability of digital health systems by health experts is to give them access to efficient software that functions reliably and that have the trusts of medical experts, for improving, thanks to user-centered design, the assistance and the expert-tool collaboration at the benefits of the patients.

To sum up, to identify biases is an essential matter when it comes to health field and specially to DHI. To limit misconception errors, but also perception ones, it is important to put in place processes to design tools and manage data to be more explicit and transparent.

Biases in algorithms

Machine Learning (ML) algorithms are now widely accepted in digital health (Gianfrancesco et al., 2018).

However, that does not prevent them from bias issues such as overreliance on automation: algorithms based on biased data, and algorithms that do not provide information that is clinically meaningful. Computer scientists and bioinformaticians, together with practitioners, biostatisticians, and epidemiologists, should outline the “intent behind the design” (Char et al., 2018), including choosing appropriate questions and settings for ML use, interpreting findings, and conducting follow-up studies. Such measures would increase the likelihood that the results of the models are meaningful and ethical and that clinical decision support tools based on these algorithms have beneficial effects.

Gianfrancesco & al. (Gianfrancesco et al., 2018) listed some recommendations to reduce biases and deficiencies in the data used by ML algorithms which may contribute to socio-economic disparities in health care : 1) Clinical decision support algorithms should also be tested for the potential introduction of discriminatory aspects throughout all stages of data processing; 2) Feedback loops should be designed to monitor and verify ML output and validity (d’Alessandro et al., 2017), ensuring that the algorithm is not correctly misinterpreting exposure-disease associations, including associations based on sex, race/ethnicity, or insurance (Cabitza et al., 2017); 3) Race/ethnicity should be captured in the electronic health record so that it can be used in models to reduce confounding and detect potential biases; 4) All variables should be used thoughtfully, however, so that the algorithms do not perpetuate disparities (C. C. Miller, 2015; O’Neil, 2016); 5) Developing ML techniques have to account for missing data and integrate external data sets that include more diverse patient populations; 6) Before analysis, data can be reviewed to ensure that they are adequately representative across racial categories and that sufficient numbers of patients who have had interruptions in their care are included; 7) Ranking the utility and quality of the information in a note or rank the importance of a note to patient care, because it enables to reduce misclassification based on implicit bias or data generated by inexperienced practitioners; 8) Fostering methods to debias ML algorithms (Bolukbasi et al., 2016), as techniques to enhance fairness and reduce indirect prejudices that result from algorithm predictions (Kamishima et al., 2012).

The future of digital health will allow to better address user factors and to transform healthcare structures (despite positive evaluations of self-management tools and automated diagnostics) as well as to enable technical aspects of service integration, such as data standards and interoperability (Huckvale et al., 2019). Indeed, the development that warrants a continued strong focus on human factors is the impact of artificial intelligence on systems of care, autonomy, and safety. For example, chatbots using ML to drive natural language computer interfaces will provide new challenges for interactions between patients and practitioners (Huckvale et al., 2019), especially in mental health. ML also brings new safety, equity, and privacy concerns. With a strong consideration in the commercial aviation industry (Rankin et al., 2016), the emergence of novel safety risks in human-computer interaction should be carefully monitored as algorithms start to be woven into everything, including discharge planning, enhanced surgical vision and ML-based decision support. To detect and address previously unanticipated equity issues, such as poor performance by biased algorithms that learn using data only from specific groups (Gianfrancesco et al., 2018), new technical strategies are necessary. Changing public attitudes around Data Governance, spurred on by topical failures in the commercial sector, may directly affect the viability of data-hungry applications, such as digital phenotyping efforts that seek to build new risk-prediction models using behavioral insights gathered automatically from smartphones and wearables (Onnela & Rauch, 2016), enabling to improve more effectively the patient-centered health outcomes, especially in mental settings.

Finally, (Naslund et al., 2015) have reviewed 44 studies that used digital technology for the treatment, diagnosis, or management of mental disorders, or for providing mental health training and education to health workers, and observed that DHI have enormous potential to improve access to health care, but vulnerable people (e.g. refugees and young people with mental disorders and relevant factors associated such as difficulties with housing or transportation) were the most forget and not included in ML strategies. Consequently, they may have insufficient information in the electronic health record to qualify for disease definitions in a clinical decision support tool that would trigger early interventions or may only be identified if their disease becomes more severe.

We thus described how to consider cognitive biases to improve diagnostic reasoning and health outcomes. It is important to be aware of some new threats such as the recent trend of social media platforms and commercial recommendation systems being used to manipulate people's inherent cognitive biases (Kido & Takadama, 2019). Studies indicate that the cognitive biases of physicians (and patients in extension) can be affected by how AI is perceived particularly at the community or social level. To improve the AI acceptability in digital health systems, understanding relationships between AI and society is very important, which includes the issues on AI and future economics or “well-being society” (such as happiness of citizen life quality) (Kido & Takadama, 2019).

Appendix.6 Anonymization and explainable AI

Anonymization is a growing research field that aims at finding efficient ways to share data all while preserving identities (Rodiya & Gill, 2015; Selvi & Pushpa, 2015). The human and ethical difficulties of privacy protection (Mittelstadt & Floridi, 2016) are exacerbated by the absence of universal standards as well as by the risk of losing information in the anonymization process (hence the anonymization vs anonymization willingness trade-off). Many thought that ML algorithms could be a solution to data privacy issues. Indeed, ML techniques are now widely accepted in digital health although deep neural networks were first thought of as a solution to data privacy issues (Gianfrancesco et al., 2018), they also are a source of concern as they are described as black boxes (Gilpin, 2018). Indeed, although their design and implementation (mathematical and computational principles) are known to developers, the reasons for their prediction and the implicit representations they encode remain unclear. Moreover, the deeper a network is, the opaquer it becomes. This lack of knowledge of the reasoning behind neural network predictions, combined with recent research that has highlighted the existence of biases in machine learning (Angwin et al., 2016) and the fragility of some algorithms (under certain conditions, it is possible to mislead a neural network for a classification task for example) (Nguyen et al., 2015), have led to general public mistrust and scientific vigilance. Beyond that, the adoption of the European Union’s new GDPR has raised concerns about the emerging tools for automated individual decision making in 2017 and the investment of the American DARPA in the XAI (eXplainable AI) project have both accelerated research on two concepts around neural network: **interpretability**, defined as a set of methods and approaches to break down all the inner mechanisms of black boxes, without necessarily understanding them (Kaadoud et al., 2019), and **explainability**, defined as the ability to fully understand the functioning of black boxes in understandable terms for humans, independently of their technical background or cognitive profile (Arrieta et al, 2020).

Research on the transparency of neural network decisions is at its infancy (Bengio et al., 2013; Lipton, 2018; Ayache et al., 2018; Gilpin et al., 2018; Guidotti et al., 2018, Arrieta et al, 2020) and a growing number of frameworks are being developed for this purpose (IBM’s AI Explainability 360 open-source library, the university of Washington’s Model-agnostic Explanations framework named LIME, Facebook’s complete library for explaining decisions made by deep network developed named Captum).To sum up, as important as it is to have powerful tools, it is even more important to try to explain and interpret them, especially in mental health.
